# Supplementary material for: A qRT-PCR Method Capable of Quantifying Specific Microorganisms Compared to NGS-Based Metagenome Profiling Data
Source: Microorganisms. 2022 Jan 30;10(2):324. doi: 10.3390/microorganisms10020324 (PMC8875016; doi:10.3390/microorganisms10020324)
Supplement: Supplementary file 1 [file microorganisms-10-00324-s001.zip › Supplementary Figure S1.pdf]

Akkermansia in silico test

Forward primer: 5’-CTTCGTGCTGGAAATCAACACC-3’

Sequences producing significant alignments

Download | New Select columns | Show 100

select all *100 sequences selected*

GenBank | Graphics | Distance tree of results | New MSA Viewer

	Description	Scientific Name	Max Score	Total Score	Query Cover	E value	Per. Ident	Acc. Len	Accession
☑	Akkermansia muciniphila strain JCM 30893 chromosome, complete genome	Akkermansia m...	44.1	74.3	100%	0.047	100.00%	2878261	CP048438.1
☑	Akkermansia muciniphila strain EB-AMDK-49 chromosome, complete genome	Akkermansia m...	44.1	74.3	100%	0.047	100.00%	2844808	CP029706.1
☑	Akkermansia muciniphila strain EB-AMDK-48 chromosome, complete genome	Akkermansia m...	44.1	74.3	100%	0.047	100.00%	2844777	CP029705.1

Reverse primer: 5’-CGATAATTCCGCTATTTTTCGC-3’

Sequences producing significant alignments

Download | New Select columns | Show 100

select all *100 sequences selected*

GenBank | Graphics | Distance tree of results | New MSA Viewer

	Description	Scientific Name	Max Score	Total Score	Query Cover	E value	Per. Ident	Acc. Len	Accession
☑	Akkermansia muciniphila strain JCM 30893 chromosome, complete genome	Akkermansia m...	46.1	46.1	100%	0.018	100.00%	2878261	CP048438.1
☑	Akkermansia muciniphila JCM 30893 DNA, complete genome	Akkermansia m...	46.1	46.1	100%	0.018	100.00%	2845645	AP021898.1
☑	Akkermansia muciniphila strain EB-AMDK-4 chromosome, complete genome	Akkermansia m...	46.1	46.1	100%	0.018	100.00%	2664010	CP024740.1

Bacteroides in silico test

Forward primer: 5'-GGTGCCTCTCAGACAATCAG-3'

Sequences producing significant alignments Download New Select columns Show 100

select all *100 sequences selected* GenBank Graphics Distance tree of results New MSA Viewer

	Description	Scientific Name	Max Score	Total Score	Query Cover	E value	Per. Ident	Acc. Len	Accession
☑	Bacteroides thetaiotaomicron strain 7330, complete genome	Bacteroides the...	40.1	40.1	100%	0.73	100.00%	6487685	CP012937.1
☑	Bacteroides ovatus strain CL06T03C20 chromosome, complete genome	Bacteroides ov...	40.1	40.1	100%	0.73	100.00%	6651367	CP072244.1
☑	Bacteroides xylanisolvens strain APCS1/XY chromosome, complete genome	Bacteroides xyl...	40.1	40.1	100%	0.73	100.00%	6461058	CP042282.1

Reverse primer: 5'-CAATGATACCACTGAATCCGCT-3'

Sequences producing significant alignments Download New Select columns Show 100

select all *96 sequences selected* GenBank Graphics Distance tree of results New MSA Viewer

	Description	Scientific Name	Max Score	Total Score	Query Cover	E value	Per. Ident	Acc. Len	Accession
☑	Bacteroides fragilis strain FDAARGOS_763 chromosome, complete genome	Bacteroides fra...	44.1	44.1	100%	0.046	100.00%	5460140	CP054003.1
☑	Bacteroides thetaiotaomicron strain 7330, complete genome	Bacteroides the...	44.1	44.1	100%	0.046	100.00%	6487685	CP012937.1
☑	Bacteroides ovatus strain CL06T03C20 chromosome, complete genome	Bacteroides ov...	44.1	44.1	100%	0.046	100.00%	6651367	CP072244.1

Bifidobacterium in silico test

Forward primer: 5'-AAGGGCATCTCCGTCAACG-3'

Sequences producing significant alignments

Download | New Select columns | Show 100

select all *100 sequences selected*

GenBank Graphics Distance tree of results New MSA Viewer

	Description	Scientific Name	Max Score	Total Score	Query Cover	E value	Per. Ident	Acc. Len	Accession
☑	Bifidobacterium breve strain JTL chromosome, complete genome	Bifidobacterium breve	38.2	38.2	100%	2.9	100.00%	2289549	CP053940.1
☑	Bifidobacterium animalis strain Probio-M8 chromosome, complete genome	Bifidobacterium animalis	38.2	38.2	100%	2.9	100.00%	1937197	CP047190.1
☑	Bifidobacterium breve strain JR01 chromosome, complete genome	Bifidobacterium breve	38.2	68.4	100%	2.9	100.00%	2304912	CP040931.1

Reverse primer: 5'-GGAGACGAAGAAGGAAGCGA-3'

Sequences producing significant alignments

Download | New Select columns | Show 50

select all *10 sequences selected*

GenBank Graphics Distance tree of results New MSA Viewer

	Description	Scientific Name	Max Score	Total Score	Query Cover	E value	Per. Ident	Acc. Len	Accession
☑	Bifidobacterium longum Jih1 DNA, complete genome	Bifidobacterium longum	40.1	70.4	100%	0.73	100.00%	2371107	AP022868.1
☑	Raphanus sativus genome assembly, chromosome: 2	Raphanus sativus	40.1	163	100%	0.73	100.00%	53721154	LR778311.1
☑	Bifidobacterium adolescentis strain ZJ2 chromosome, complete genome	Bifidobacterium adole...	40.1	40.1	100%	0.73	100.00%	2401766	CP047129.1

Phascolarctobacterium in silico test

Forward primer: 5'-TTCCTGGTTATGTGCTTGTAGAG-3'

Sequences producing significant alignments

Download | New Select columns | Show 100

select all *100 sequences selected*

GenBank | Graphics | Distance tree of results | New MSA Viewer

	Description	Scientific Name	Max Score	Total Score	Query Cover	E value	Per. Ident	Acc. Len	Accession
☑	Rodentibacter pneumotropicus strain NCTC8284 genome assembly, chromosome: 1	Rodentibacter p...	46.1	46.1	100%	0.018	100.00%	2439769	LR134405.1
☑	Phascolarctobacterium faecium isolate Phascolarctobacterium succinatutens 82G5 genome assembly,...	Phascolarctoba...	46.1	46.1	100%	0.018	100.00%	2550003	LR215982.1
☑	Phascolarctobacterium faecium JCM 30894 DNA, complete genome	Phascolarctoba...	46.1	46.1	100%	0.018	100.00%	2454371	AP019004.1

Reverse primer: 5'-CAGTCAAAGGAATCGGTTTAGTA-3'

Sequences producing significant alignments

Download | New Select columns | Show 100

select all *100 sequences selected*

GenBank | Graphics | Distance tree of results | New MSA Viewer

	Description	Scientific Name	Max Score	Total Score	Query Cover	E value	Per. Ident	Acc. Len	Accession
☑	Phascolarctobacterium faecium isolate Phascolarctobacterium succinatutens 82G5 genome assembly,...	Phascolarctoba...	46.1	46.1	100%	0.018	100.00%	2550003	LR215982.1
☑	Phascolarctobacterium faecium JCM 30894 DNA, complete genome	Phascolarctoba...	46.1	46.1	100%	0.018	100.00%	2454371	AP019004.1
☑	Phascolarctobacterium faecium strain G104 chromosome, complete genome	Phascolarctoba...	46.1	46.1	100%	0.018	100.00%	2364938	CP061002.1

Roseburia in silico test

Forward primer: 5'-AAATACCCGTGGTGTTACCG-3'

Sequences producing significant alignments Download New Select columns Show 100

select all *100 sequences selected* GenBank Graphics Distance tree of results New MSA Viewer

	Description	Scientific Name	Max Score	Total Score	Query Cover	E value	Per. Ident	Acc. Len	Accession
☑	Roseburia intestinalis L1-82 genome assembly, chromosome: 1	Roseburia intes...	40.1	40.1	100%	0.73	100.00%	4493348	LR027880.1
☑	Acetobacterium sp. KB-1 chromosome	Acetobacterium...	40.1	40.1	100%	0.73	100.00%	3988368	CP030040.1
☑	Acetobacterium woodii DSM 1030, complete genome	Acetobacterium...	40.1	40.1	100%	0.73	100.00%	4044777	CP002987.1
☑	Roseburia hominis isolate MGYG-HGUT-02517 genome assembly, chromosome: 1	Roseburia homi...	40.1	40.1	100%	0.73	100.00%	3592125	LR699011.1
☑	Roseburia intestinalis XB6B4 draft genome	Roseburia intes...	40.1	40.1	100%	0.73	100.00%	4286292	FP929050.1
☑	Roseburia intestinalis M50/1 draft genome	Roseburia intes...	40.1	40.1	100%	0.73	100.00%	4143550	FP929049.1

Reverse primer: 5'-GTGTCTCCCTCTGTAAAGTCA-3'

Sequences producing significant alignments Download New Select columns Show 100

select all *100 sequences selected* GenBank Graphics Distance tree of results New MSA Viewer

	Description	Scientific Name	Max Score	Total Score	Query Cover	E value	Per. Ident	Acc. Len	Accession
☑	Roseburia intestinalis L1-82 genome assembly, chromosome: 1	Roseburia intes...	42.1	42.1	100%	0.18	100.00%	4493348	LR027880.1
☑	Roseburia intestinalis XB6B4 draft genome	Roseburia intes...	42.1	42.1	100%	0.18	100.00%	4286292	FP929050.1
☑	Roseburia intestinalis M50/1 draft genome	Roseburia intes...	42.1	42.1	100%	0.18	100.00%	4143550	FP929049.1

Figure S1. In silico test to confirm of primer specificity for targeted bacterial genus.
