# Supplementary material for: A qRT-PCR Method Capable of Quantifying Specific Microorganisms Compared to NGS-Based Metagenome Profiling Data
Source: Microorganisms. 2022 Jan 30;10(2):324. doi: 10.3390/microorganisms10020324 (PMC8875016; doi:10.3390/microorganisms10020324)
Supplement: Supplementary file 1 [file microorganisms-10-00324-s001.zip › Supplementary Figure S2.pdf]

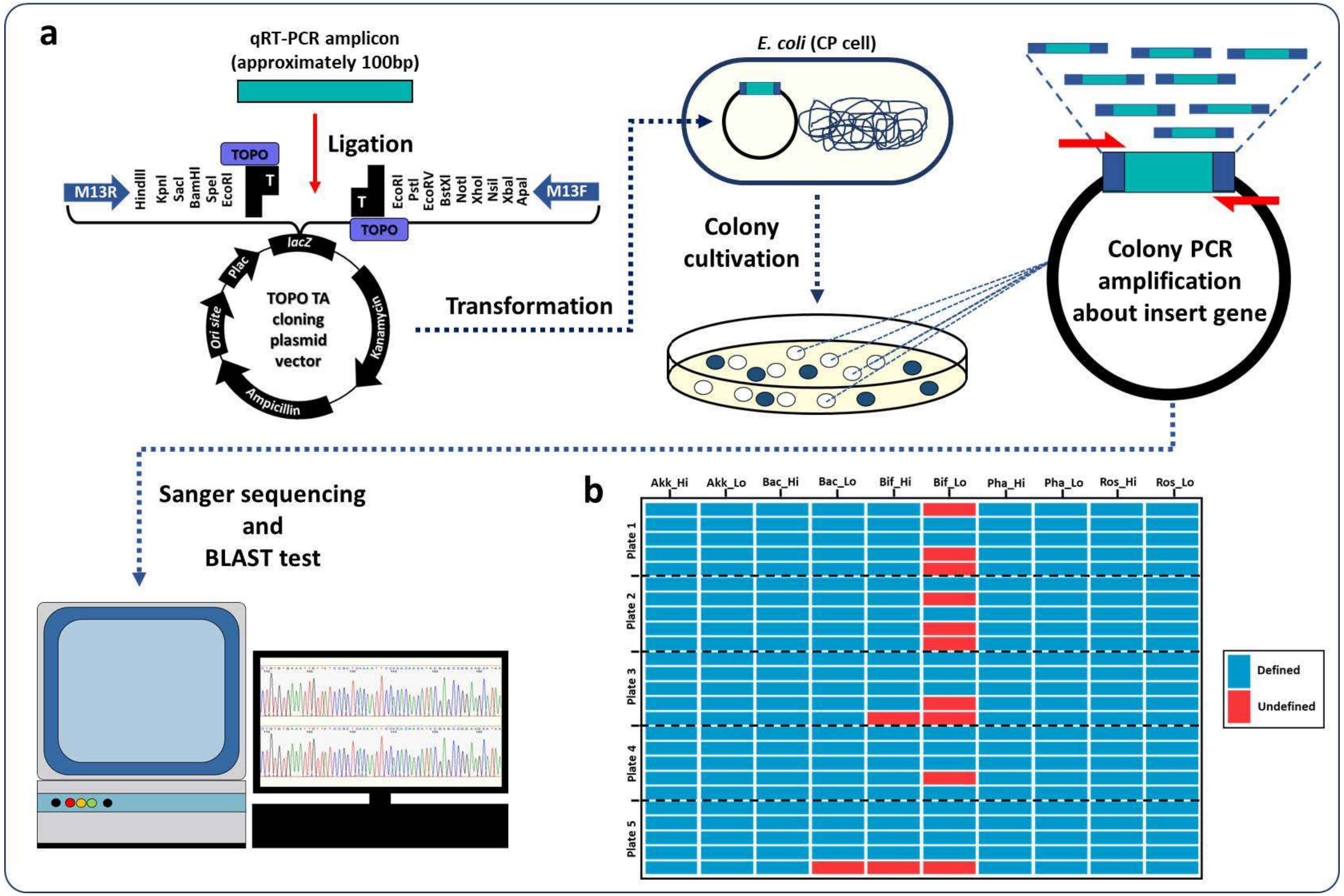

Figure S2. Verification of the specificity and accuracy of primers for quantifying the five bacterial genera using Sanger sequencing.
